# *Bifidobacterium animalis* subsp. *lactis* BL-G101 Protects Against UVB-Induced Photoaging in HS68 Dermal Fibroblasts

**DOI:** 10.3390/nu18183013

**Published:** 2026-09-15

**Authors:** Jingya Guo, Ruixuan Geng, Seong-Gook Kang, Kunlun Huang, Tao Tong

**Affiliations:** 1 College of Food Science and Nutritional Engineering, China Agricultural University, Beijing 100083, China; 2 Department of Nutrition and Food Hygiene, School of Public Health, Dalian Medical University, No. 9 West Section Lvshun South Road, Dalian 116044, China; 3 Department of Food Engineering and Solar Salt Research Center, Mokpo National University, Muangun 58554, Republic of Korea; 4 Key Laboratory of Safety Assessment of Genetically Modified Organism (Food Safety), Ministry of Agriculture, Beijing 100083, China; 5 Beijing Laboratory for Food Quality and Safety, Beijing 100083, China

**Keywords:** *Bifidobacterium animalis* subsp. *lactis* BL-G101, skin photoaging, dermal fibroblasts

## Abstract

**Background**: Skin photoaging is primarily caused by prolonged ultraviolet (UV) exposure, which manifests as dryness and wrinkle formation. Recent studies highlight that beneficial microorganisms have garnered considerable attention for their emerging role in modulating host health, especially in skin photoaging. **Methods**: To investigate the role of two strains, *Bifidobacterium animalis* subsp. *lactis* BL-G101 (BL-G101) and *Lactobacillus acidophilus* LA-G80 (LA-G80), in UVB-induced photoaging, we conducted experiments on human Hs68 dermal fibroblasts. Using untargeted metabolomics, we explored the potential mechanisms underlying BL-G101 treatment in Hs68 cells by profiling differential metabolites in the intracellular and extracellular metabolomes. **Results**: Our results demonstrated that BL-G101 significantly inhibited UVB-induced reduction in collagen and hyaluronic acid (HA), whereas LA-G80 showed no notable effects. BL-G101 treatment also protects against UVB-induced oxidative stress, which was evidenced by significantly decreased MDA levels and enhanced SOD activity. Moreover, BL-G101 suppressed UVB-induced inflammation by significantly decreasing IL-1β and TNF-α levels. SA-β-gal staining revealed a reduction in senescent fibroblasts following BL-G101 treatment. Untargeted metabolomics suggested that the anti-photoaging effects of BL-G101 may involve the modulation of metabolic pathways of biosynthesis of cofactors, glycine, serine and threonine metabolism, and arginine biosynthesis. **Conclusions**: These preclinical findings demonstrate that BL-G101 reduces dermal photoaging, supporting its potential as a functional food ingredient against skin aging.

## 1. Introduction

As the largest organ of the human body, the skin is directly exposed to the external environment [1]. Skin aging results from both internal factors (age and genetics) and several external factors, which include ultraviolet (UV) radiation, smoking, chemical exposure, and atmospheric pollution [2]. Among these factors, chronic UV radiation exposure-induced skin photoaging is the most prevalent form of extrinsic skin aging, accounting for over 80% of facial aging [3,4]. The visible manifestations of photoaging encompass wrinkles, skin roughness, hyperpigmentation, and deterioration in skin elasticity [5]. In addition to affecting the skin’s appearance, photoaging also predisposes individuals to various dermatological pathologies, such as actinic keratosis, basal cell carcinoma, squamous cell carcinoma, and melanoma [6]. These diseases also pose a significantly higher risk among the population in high-altitude areas [7]. Given the public health burden of photoaging and associated disorders, developing effective interventions and elucidating their mechanisms is a research priority.

For the past few decades, probiotics have been increasingly recognized, typically described as live microorganisms that “when administered in adequate amounts, confer a health benefit on the host” [8], according to the World Health Organization. Probiotics are known to restore intestinal microbial balance and modulate immune responses. Recent preclinical and clinical studies have also indicated that probiotics could mitigate skin inflammation and oxidative stress, enhance the skin’s barrier function, and protect against UV damage [9]. Currently, probiotics have been regarded as an excellent potential treatment avenue for skin photoaging [10].

*Bifidobacterium animalis* and *Lactobacillus acidophilus* are widely used for treating gastrointestinal disorders such as inflammatory bowel disease and enhancing immune function [11,12]. Notably, both strains are recognized as safe for use in foods in China, being included in the National Health Commission’s “List of Microorganisms Permitted for Use in Foods.” In recent years, these strains have been recognized for their additional benefits, particularly in improving skin health. In a randomized, placebo-controlled, triple-blinded study enrolling 148 female volunteers, consumption of *B. animalis* subsp. *lactis* BL-04 for 4 weeks led to significant improvements in facial skin wrinkle parameters, including wrinkle area, volume, average depth, and arithmetic average roughness [13]. Studies have shown that *B. animalis* subsp. *lactis* BX-BC08 was capable of regulating the gut microbiota, secreting alpha-ketoglutaric acid, and alleviating atopic dermatitis (AD) in a murine MC903-induced model [14]. Moreover, several strains of *L. acidophilus*, including IDCC 3302, KCCM12625P, and L-55, have demonstrated beneficial effects on skin health. Specifically, *L. acidophilus* IDCC 3302 exhibits antiphotoaging, antiwrinkle, and moisturizing effects in a UVB-induced hairless mouse model [15]. *L. acidophilus* KCCM12625P has been found to reduce wrinkle formation and inhibit melanogenesis in cells [16], while *L. acidophilus* L-55 inhibits the progression of AD-like lesions in NC/Nga mice [17].

Although studies have shown that certain probiotic strains could alleviate skin photoaging, the roles of *Bifidobacterium animalis* subsp. *lactis* BL-G101 (BL-G101) and *Lactobacillus acidophilus* LA-G80 (LA-G80) in photoaging are still unclear. Therefore, the present study was designed to investigate the potential anti-photoaging effects of BL-G101 and LA-G80 using an in vitro model of human Hs68 dermal fibroblasts. To elucidate the underlying mechanisms, an untargeted metabolomics approach was performed to identify differential metabolites in the intracellular as well as extracellular metabolomes of Hs68 dermal fibroblasts.

## 2. Materials and Methods

### 2.1. Reagents

Dulbecco’s Modified Eagle Medium (DMEM, #C11995500BT), penicillin-streptomycin solution (#15140-122), fetal bovine serum (#10270-106), and 0.25% trypsin-ethylene diamine tetraacetic acid solution (#25200-056) were purchased from Gibco (Thermo Fisher Scientific, Bohemia, NY, USA). The Cell Counting Kit-8 (#C0037), Senescence β-Galactosidase Staining Kit (#C0602), and Superoxide Dismutase (SOD) Assay Kit (#S0101S) were procured from Beyotime (Shanghai, China). The Malondialdehyde (MDA) Assay Kit (BC0025) was procured from Solarbio (Beijing, China). The Human TNF-α ELISA Kit (#EH02) and Human IL-1β ELISA Kit (#EH18) were supplied by Biokits Technologies (Beijing, China). The Human Pro-Collagen Iα1 ELISA Kit (#EK1C01) was from MultiSciences Biotech (Hangzhou, China), and the Human hyaluronic acid (HA) ELISA Kit (#CSB-E04805h) was provided by CUSABIO (Wuhan, China).

### 2.2. Strains

The BL-G101 and LA-G80 used in this work were provided by BioGrowing Co., Ltd. (Shanghai, China). For the in vitro studies, both strains were resuspended in PBS (pH 7.2).

### 2.3. Cell Culture

The human dermal fibroblast cell line Hs68 was obtained from the American Type Culture Collection (Manassas, VA, USA). Culture maintenance was performed in DMEM containing 10% FBS and 1% penicillin-streptomycin, with incubation at 37 °C under a 5% CO_2_ atmosphere. For bacterial treatment, the culture medium was replaced with antibiotic-free DMEM before the addition of BL-G101 or LA-G80. For subculturing, cells underwent PBS washing followed by detachment using a 0.25% trypsin/ethylenediaminetetraacetic acid solution.

### 2.4. UVB Irradiation

According to previous reports [5,18], UVB irradiation was performed as previously described. Briefly, a UVB lamp (TL 20W/12 RS SLV/25, Philips, The Netherlands) was positioned 30 cm above the cells, providing an irradiance of 0.126 mW/cm^2^, which was measured using a UVB irradiance meter. During irradiation, cells were maintained in 50 μL PBS. The final UVB dose was 5 mJ/cm^2^.

### 2.5. Cell Viability Assay

Determination of cell viability relied on the CCK-8 assay according to a previous description [19]. Briefly, 96-well plates were seeded with cells that were then cultured for 24 h. Subsequent treatment with BL-G101 or LA-G80 (probiotic concentrations of 10^3^–10^8^ CFU/mL) lasted another 24 h. Control samples included untreated cells (0 CFU/mL) and a blank (medium only). Following treatment, CCK-8 solution (10 µL per well) was added and incubated for 3 h. The absorbance at 450 nm was recorded, enabling calculation of cell viability.

### 2.6. Determination of Type I Collagen and HA Levels

Hs68 cells were plated into 12-well plates at a density of 5 × 10^5^ cells per well and incubated for 24 h. Following UVB exposure, the cells received a 24 h treatment with the probiotic strains and were then harvested. The supernatants were assessed for type I collagen and HA levels using ELISA kits in line with the manufacturers’ instructions, with absorbance measured at 450 nm. These measured values were subsequently adjusted to the total cellular protein content for final normalization.

### 2.7. Determination of MDA and SOD Levels

Cell treatment followed the procedures described in Section 2.6. For lipid peroxidation evaluation, MDA was measured using the thiobarbituric acid reaction, whose adduct product shows absorbance at 532 nm and 600 nm. SOD activity was assessed with a commercial kit employing the WST-8 method, and the absorbance was recorded at 450 nm.

### 2.8. Determination of IL-1β and TNF-α Levels

Cells treated as described in Section 2.6. The concentrations of IL-1β and TNF-α were measured quantitatively using an ELISA assay, with the procedure following the manufacturer’s instructions, and normalized to total cellular protein content.

### 2.9. Senescence-Associated β-Galactosidase (SA-β-Gal) Staining

Cells were processed according to the kit’s instructions: fixation in fixative solution for 15 min, PBS washing, and then addition of 1 mL of freshly prepared SA-β-gal staining solution (10 µL β-galactosidase-stained solutions A and B, 930 µL solution C, and 50 µL X-gal solution). After an overnight dark incubation at 37 °C, the cells were rinsed with PBS for 10 min. SA-β-Gal-positive cells were viewed under a NIKON ECLIPSE Ci-L upright microscope.

### 2.10. Untargeted Metabolomics Analysis

#### 2.10.1. Sample Preparation

Sample preparation: For untargeted metabolomics analysis, Hs68 dermal fibroblasts were divided into three groups: the control, UVB, and BL-G101 treatment groups. Cells were cultured in 6-well plates and treated as described in Section 2.3 and Section 2.4. At the end of the treatment period, culture supernatants were collected for extracellular metabolite analysis, while the cells were harvested and centrifuged to obtain cell pellets for intracellular metabolite analysis. The collected samples were subsequently processed as described below.

Extracellular metabolites: Culture supernatant (100 µL) was combined with 400 µL of acetonitrile: methanol (1:1, *v*/*v*) spiked with 0.02 mg/mL internal standard. The mixture underwent vortex mixing (30 s) and ultrasonication (5 °C, 40 kHz, 30 min), then protein precipitation was achieved by a 30 min incubation at −20 °C. After centrifugation (13,000× *g*, 4 °C, 15 min), the clear upper phase was evaporated to dryness under nitrogen. The dry residue was redissolved in 100 µL of acetonitrile: water (1:1, *v*/*v*) via ultrasonication (5 °C, 40 kHz, 5 min), followed by another centrifugation (13,000× *g*, 4 °C, 10 min). The resulting supernatant was loaded into an injection vial for LC-MS/MS analysis.

Intracellular metabolites: Following removal of the culture medium, wash the cell pellet twice with PBS. Subsequently, samples were resuspended in 400 µL of extraction solvent (methanol: water = 4:1, *v*/*v*) containing 0.02 mg/mL L-2-chlorophenylalanine as the internal standard. The suspension was homogenized using a frozen tissue grinder (−10 °C, 50 Hz, 6 min) and then extracted ultrasonically (5 °C, 40 kHz, 30 min). The homogenate was kept at −20 °C for 30 min, followed by centrifugation (13,000× *g*, 4 °C, 15 min). The resultant supernatant was collected for LC-MS/MS analysis.

#### 2.10.2. LC-MS Analysis

Chromatographic separation was conducted on a Thermo UHPLC-Q Exactive HF-X system coupled with an ACQUITY HSS T3 column (100 mm × 2.1 mm i.d., 1.8 µm; Waters, Milford, MA, USA) at Majorbio Bio-Pharm Technology Co. Ltd. (Shanghai, China). Mobile phases consisted of (A) water: acetonitrile (95:5, *v*/*v*) containing 0.1% formic acid and (B) acetonitrile: isopropanol: water (47.5:47.5:5, *v*/*v*/*v*) containing 0.1% formic acid. The flow rate was set at 0.40 mL/min, and the column temperature was maintained at 40 °C. Mass spectrometric data were acquired in both positive and negative electrospray ionization (ESI) modes on the Thermo UHPLC-Q Exactive HF-X Mass Spectrometer. The capillary voltages were set at 3500 V and 2800 V in positive and negative ion modes, respectively. The sheath gas and auxiliary gas were set at 40 psi and 10 psi, respectively, and the ion source temperature was maintained at 400 °C. Normalized collision energy, 20–40–60 V rolling for MS/MS. The full MS scan resolution was 60,000, and the MS/MS resolution was 7500. Data acquisition was performed in data-dependent acquisition (DDA) mode over a mass range of *m*/*z* 70 to 1050.

Raw LC/MS data were processed using Progenesis QI software (version 3.0, Waters, USA) to generate a three-dimensional data matrix containing sample information, metabolite names, and peak intensity. Internal standard peaks and known false positives were removed. Metabolite identification was performed by searching against HMDB, Metlin, and the Majorbio database. The resulting data matrix was uploaded to the Majorbio cloud platform. Features detected in less than 80% of samples were filtered out. Missing values were imputed with the minimum metabolite intensity, and data were normalized by the sum of metabolite intensities. QC samples with relative standard deviation > 30% were excluded, and the data were log_10_-transformed before statistical analysis. Partial least squares discriminant analysis (PLS-DA) was performed using the R package “ropls” (version 1.6.2) with 7-fold cross-validation. Differential metabolites were selected based on variable importance in projection (VIP) > 1 from the OPLS-DA model and *p* < 0.05. Pathway enrichment and topology analysis were performed using the KEGG database.

### 2.11. Statistical Analysis

All data are presented as mean ± standard error of the mean (SEM). Statistical analyses were conducted using one-way ANOVA with Dunnett’s post hoc test, and all analyses were performed with GraphPad Prism (version 9.0, GraphPad Software, Inc., San Diego, CA, USA). Differences were considered statistically significant at *p* < 0.05.

## 3. Results

### 3.1. BL-G101 and LA-G80 Have No Effect on Human Dermal Fibroblast Viability

**Figure 1:**
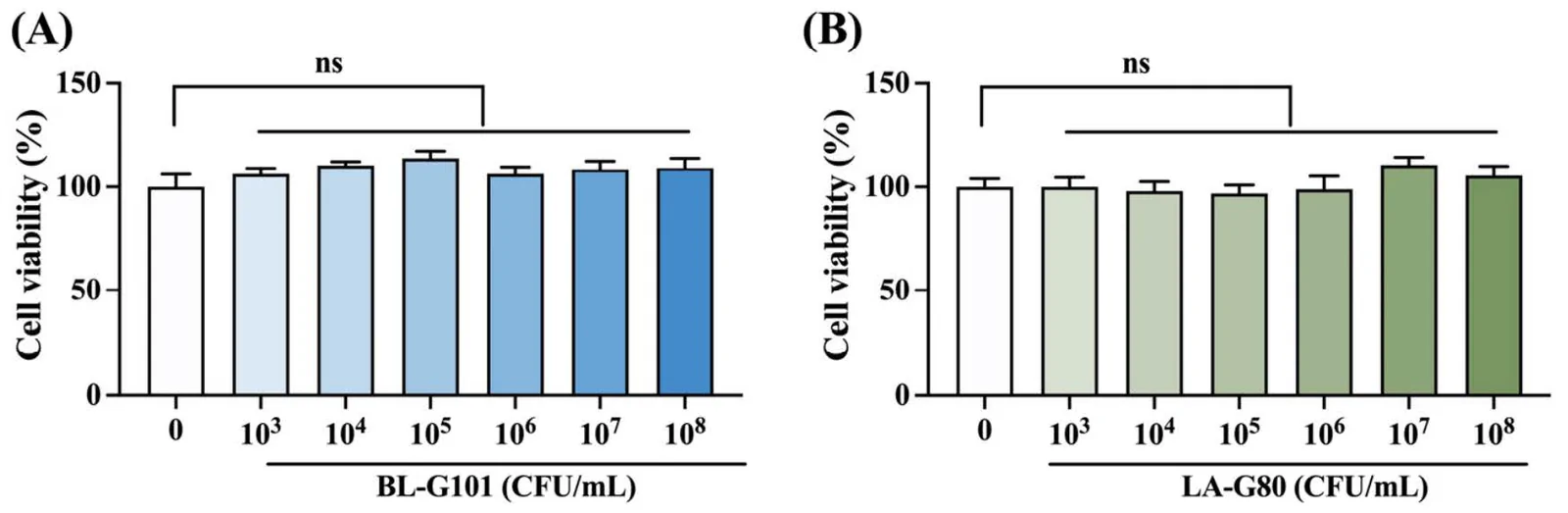
Effects of BL-G101 and LA-G80 on the cell viability of Hs68 human dermal fibroblasts. (**A**) Cells treated with BL-G101. (**B**) Cells treated with LA-G80. Data are expressed as means ± SEM (*n* = 6). ns, no significant difference (*p* > 0.05).

We assessed the effects of BL-G101 and LA-G80 exposure for 24 h on cell viability using the CCK8 assay in human dermal fibroblasts. At concentrations up to 10^8^ CFU/mL, neither BL-G101 nor LA-G80 influenced the viability of human dermal fibroblasts (Figure 1A,B). Accordingly, a concentration of 10^8^ CFU/mL was selected for both BL-G101 and LA-G80 in subsequent experiments.

### 3.2. BL-G101 Mitigates UVB-Induced Reductions in Collagen and HA in Human Hs68 Dermal Fibroblasts

**Figure 2:**
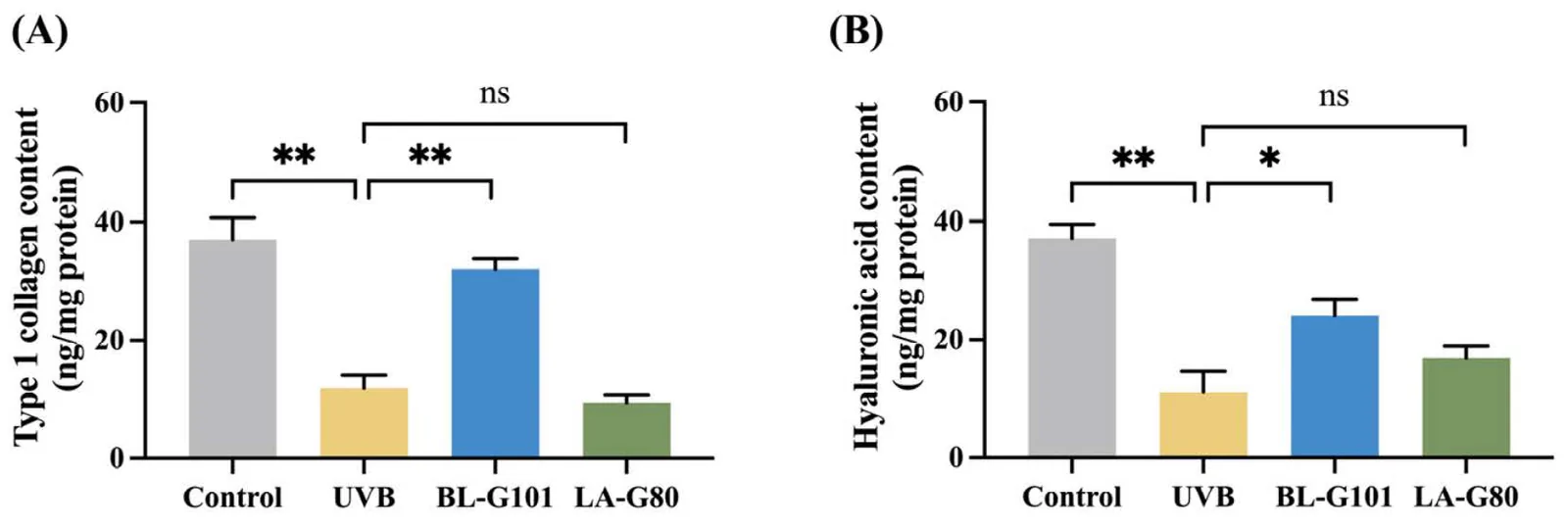
Effects of BL-G101 and LA-G80 on the levels of type I procollagen and HA in cells. (**A**) Type I procollagen level. (**B**) HA level. Data are expressed as means ± SEM (*n* = 3). Significant differences between groups are indicated by asterisks: * *p* < 0.05 and ** *p* < 0.01 versus the UVB group; ns, not significant (*p* > 0.05) versus the UVB group.

To evaluate the protection of BL-G101 and LA-G80 against UVB-induced photoaging damage, we measured type I procollagen and HA levels using ELISA kits in human dermal fibroblasts. The results demonstrated a significant reduction in the levels of type I procollagen and HA in UVB-exposed cells compared to control cells (Figure 2A,B), demonstrating that UVB irradiation induces skin photoaging damage. Treatment with BL-G101 significantly elevated the levels of type I procollagen and HA, whereas LA-G80 had no significant effect (Figure 2A,B). Since collagen and HA are the main components of the dermal ECM, restoration of their levels is recognized to effectively ameliorate skin photoaging. These findings indicate that BL-G101 effectively alleviates UVB-induced damage; thus, subsequent experiments were all conducted using BL-G101.

### 3.3. BL-G101 Alleviates UVB-Induced Oxidative Stress in Human Hs68 Dermal Fibroblasts

**Figure 3:**
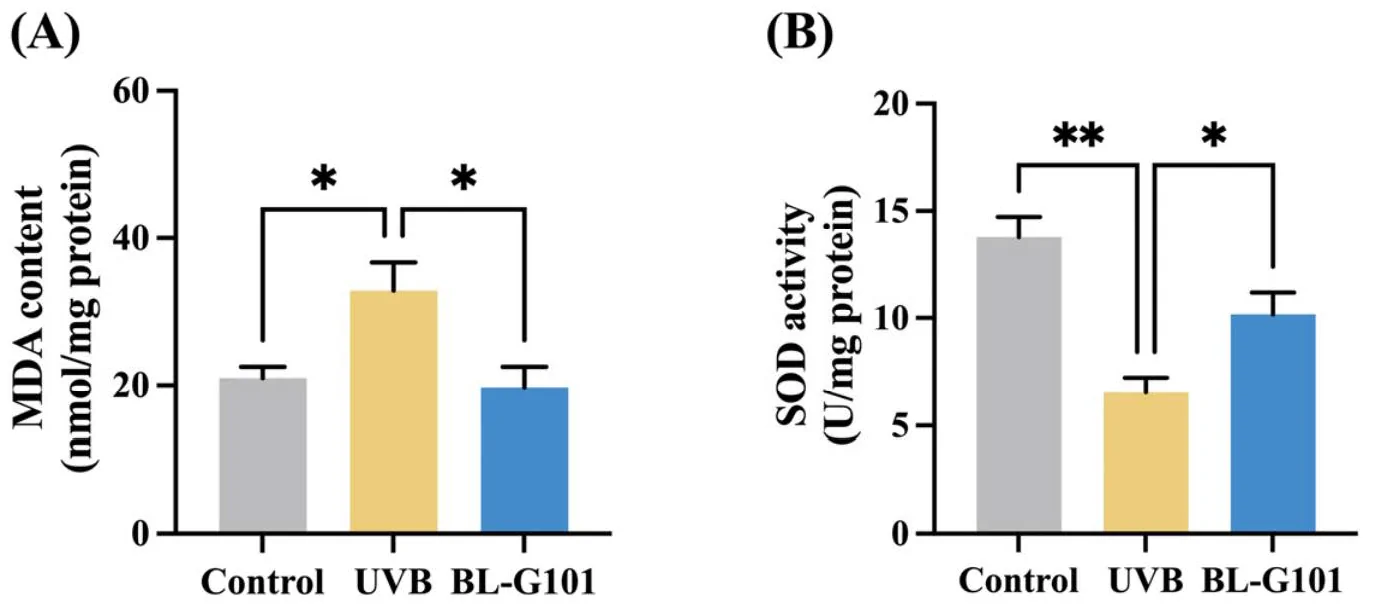
BL-G101 inhibits UVB-induced oxidative stress in Hs68 cells. (**A**) MDA level. (**B**) SOD activity. Data are expressed as means ± SEM (*n* = 3). Asterisks indicate significant differences between groups: * *p* < 0.05 and ** *p* < 0.01 versus the UVB group.

We next assessed the effect of BL-G101 treatment on intracellular oxidative stress in UVB-irradiated Hs68 dermal fibroblasts. Compared with untreated controls, UVB exposure led to a significant increase in MDA levels and a decrease in SOD activity (Figure 3A,B). The BL-G101 treatment markedly reduced MDA accumulation and increased SOD activity (Figure 3A,B). Collectively, these results indicate that BL-G101 alleviates oxidative stress induced by UVB in human Hs68 cells.

### 3.4. BL-G101 Suppresses UVB-Induced Inflammation in Human Hs68 Fibroblasts

**Figure 4:**
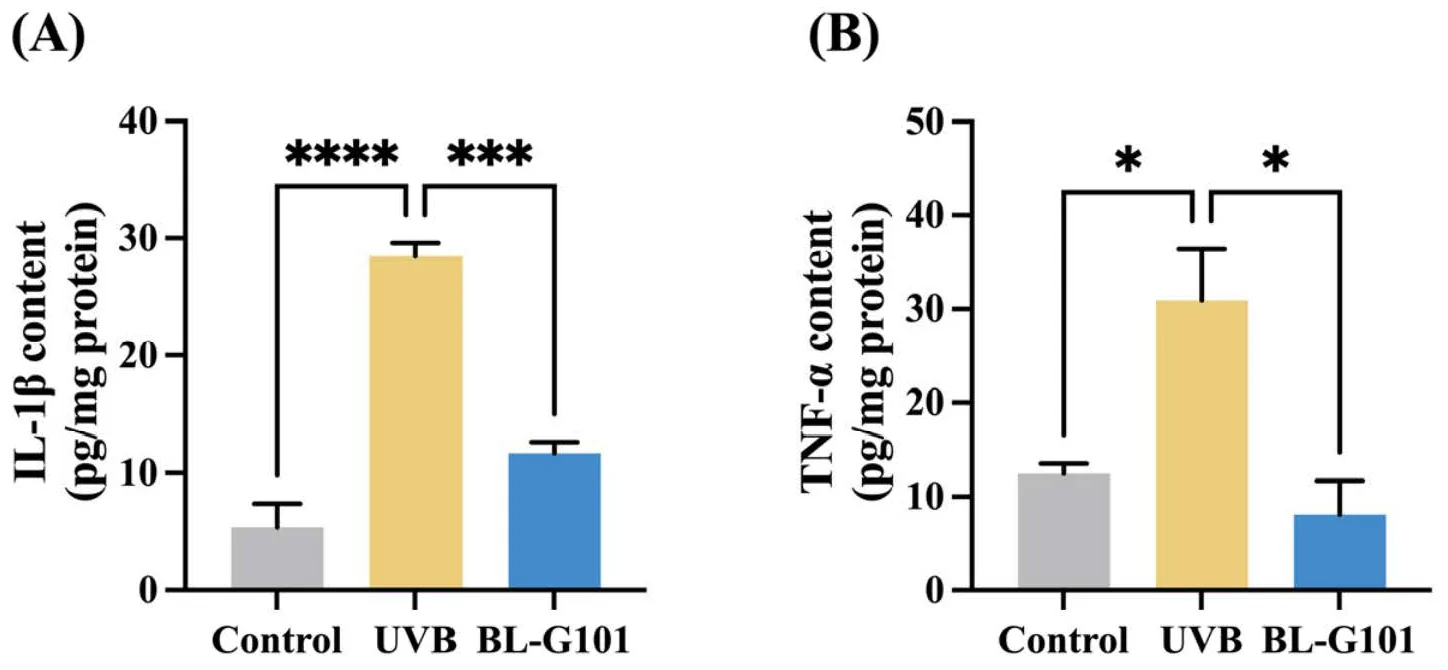
BL-G101 suppresses UVB-induced inflammation in Hs68 cells. (**A**) IL-1β level. (**B**) TNF-α level. Data are expressed as means ± SEM (*n* = 3). Asterisks indicate significant differences between groups: * *p* < 0.05, *** *p* < 0.001, and **** *p* < 0.0001 versus the UVB group.

To evaluate the anti-inflammatory effects of the BL-G101, the levels of TNF-α and IL-1β, two proinflammatory cytokines involved in skin photoaging, were measured. As shown in Figure 4, UVB irradiation significantly increased IL-1β and TNF-α levels, whereas BL-G101 treatment significantly reduced both cytokine levels compared to the UVB-irradiated group (Figure 4A,B). These findings demonstrate that BL-G101 reduces UVB-induced inflammation in human Hs68 cells.

### 3.5. BL-G101 Inhibits UVB-Induced Accumulation of Senescent Fibroblasts

**Figure 5:**
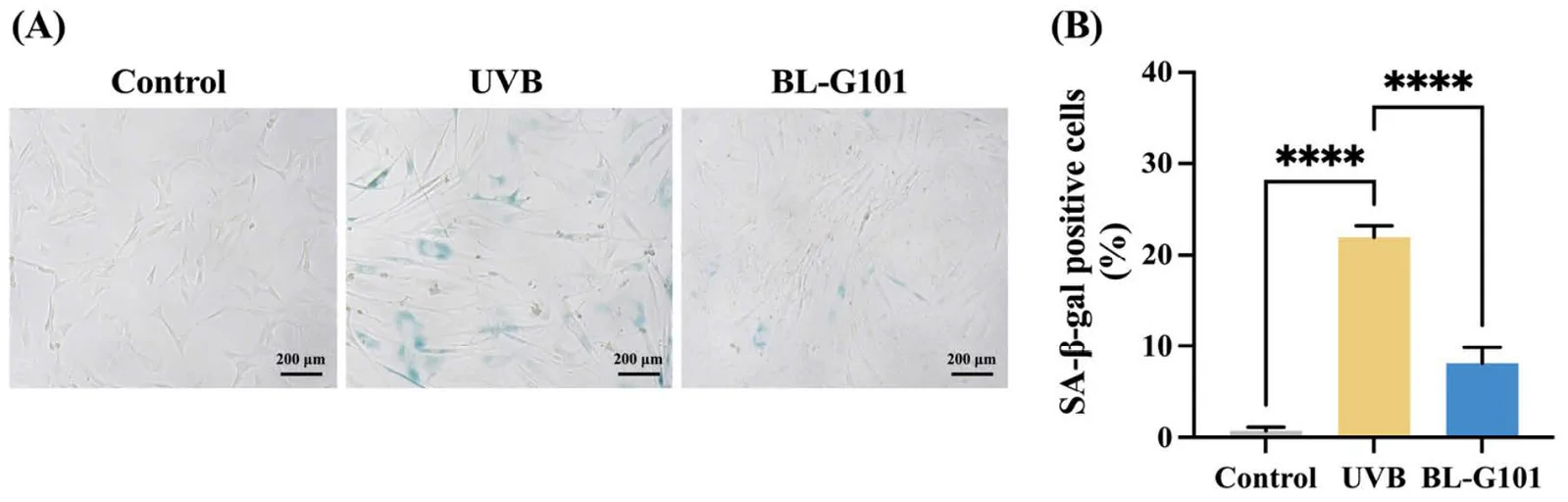
BL-G101 reduces UVB-induced accumulation of senescent fibroblasts in Hs68 cells. (**A**) SA-β-gal staining. Scale bars: 200 µm. (**B**) Percentage of SA-β-gal-positive cells. Data are expressed as means ± SEM (*n* = 9). Significant differences between groups are indicated by asterisks: **** *p* < 0.0001 versus the UVB group.

During the aging process, senescent cells accumulate in the skin [20]. To examine whether BL-G101 inhibits the accumulation of senescent cells, we performed SA-β-gal staining on Hs68 dermal fibroblasts following UVB exposure. The results showed that, compared with untreated controls, UVB irradiation significantly elevated the number of SA-β-gal-positive cells (Figure 5A). Treatment with BL-G101 significantly reduced the number of SA-β-Gal-positive cells (Figure 5A). Quantitative analysis further confirmed that the proportion of senescent cells decreased significantly following treatment with BL-G101 (Figure 5B). These findings indicate that BL-G101 reduces the accumulation of UVB-induced senescent fibroblasts.

### 3.6. PLS-DA of Intracellular and Extracellular Metabolic Profiles

**Figure 6:**
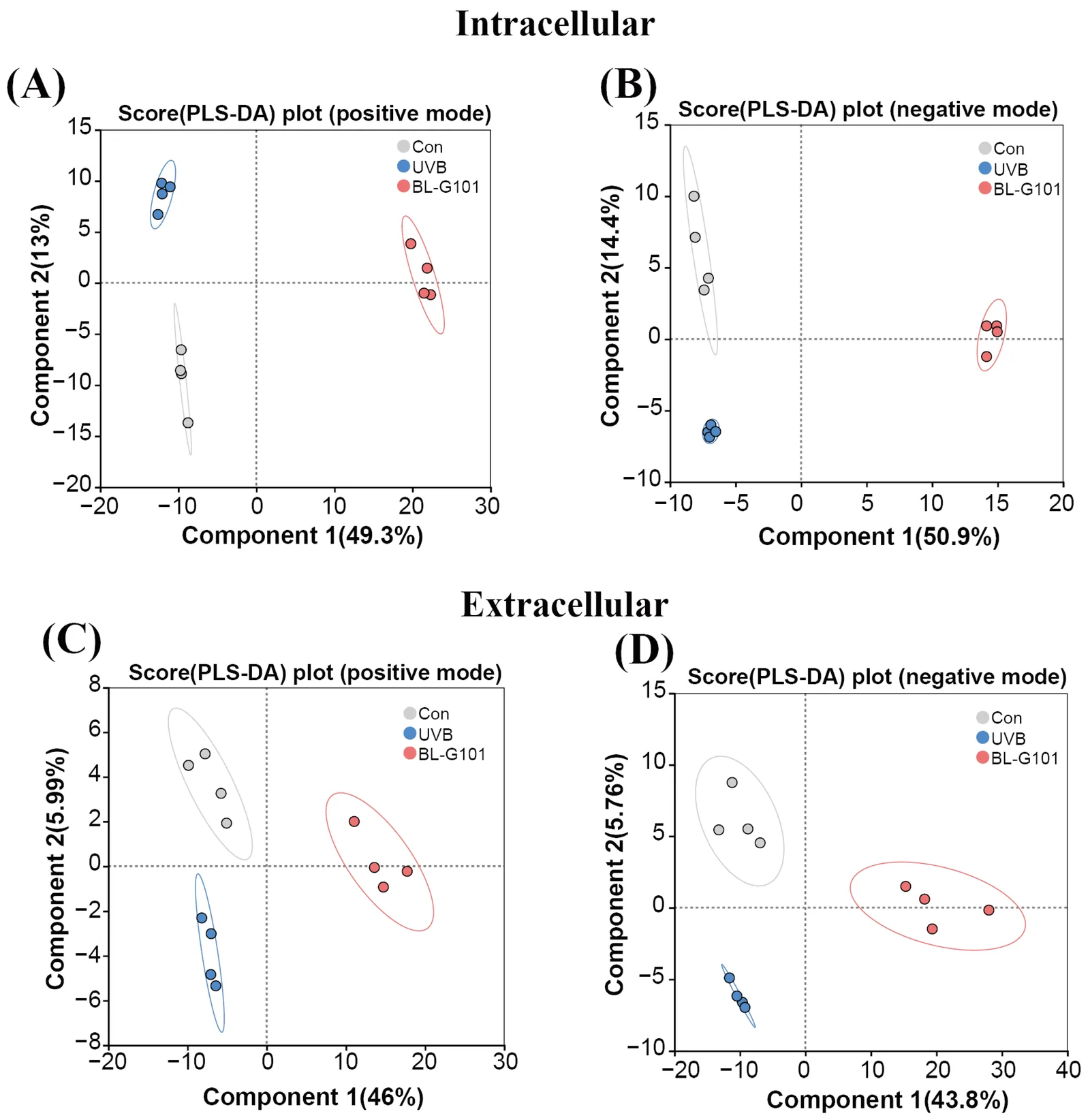
PLS-DA score plots of intracellular and extracellular metabolomic profiles. (**A**) Intracellular metabolome in positive ion mode. (**B**) Intracellular metabolome in negative ion mode. (**C**) Extracellular metabolome in positive ion mode. (**D**) Extracellular metabolome in negative ion mode.

To explore the potential mechanisms underlying the protective effects of BL-G101 against UVB-induced photoaging, untargeted metabolomics was performed on both the intracellular and extracellular levels of Hs68 dermal fibroblasts. PLS-DA was performed to distinguish samples from the control, UVB, and BL-G101 groups. For intracellular metabolites, clear separation among the three groups was observed in both positive and negative ionization modes (Figure 6A,B). Similar separation patterns were also observed in the extracellular metabolites, with the three groups distinctly separated (Figure 6C,D). These results indicate that BL-G101 treatment induces obvious alterations in both the intracellular and extracellular metabolomic profiles of UVB-exposed Hs68 cells.

### 3.7. Identification and Classification of Differential Metabolites

**Figure 7:**
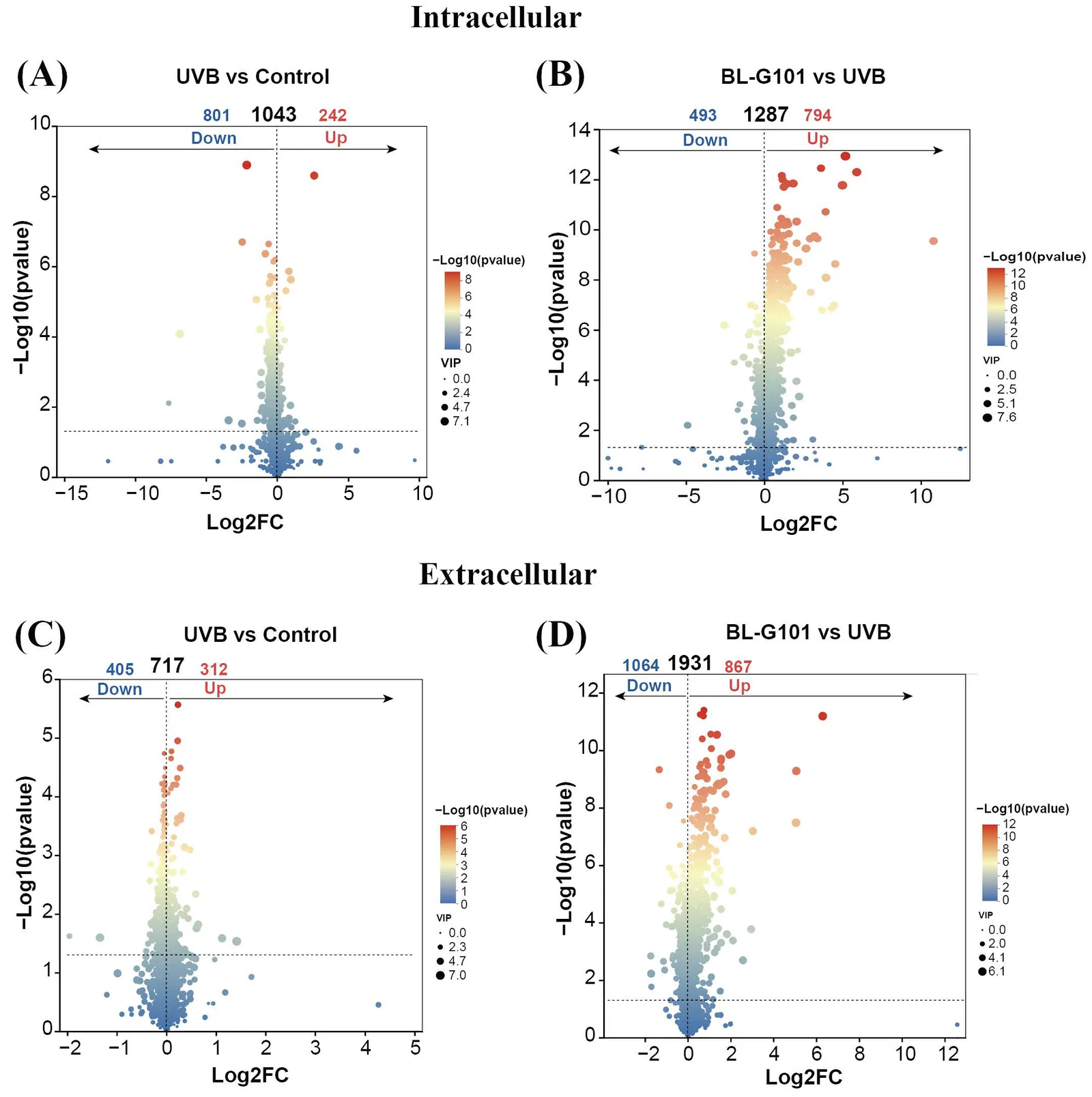
Volcano plots of intracellular and extracellular differential metabolites following BL-G101 treatment in Hs68 cells. (**A**) Intracellular differential metabolites between the UVB and control groups. (**B**) Intracellular differential metabolites between BL-G101 and UVB groups. (**C**) Extracellular differential metabolites between the UVB and control groups. (**D**) Extracellular differential metabolites between BL-G101 and UVB groups. The horizontal dashed line indicates the statistical significance threshold of *p* < 0.05, and the vertical dashed line indicates log_2_FC = 0, separating upregulated and downregulated metabolites.

In the intracellular metabolome, UVB exposure resulted in 1043 differential metabolites relative to the control group (801 downregulated, 242 upregulated) (Figure 7A). BL-G101 treatment yielded 1287 differential metabolites compared with the UVB group (493 downregulated, 794 upregulated) (Figure 7B). For the extracellular metabolome, 717 metabolites differed between the UVB and control groups (405 downregulated, 312 upregulated) (Figure 7C), while BL-G101 treatment produced 1931 differential metabolites relative to the UVB group (1064 downregulated, 867 upregulated) (Figure 7D).

**Figure 8:**
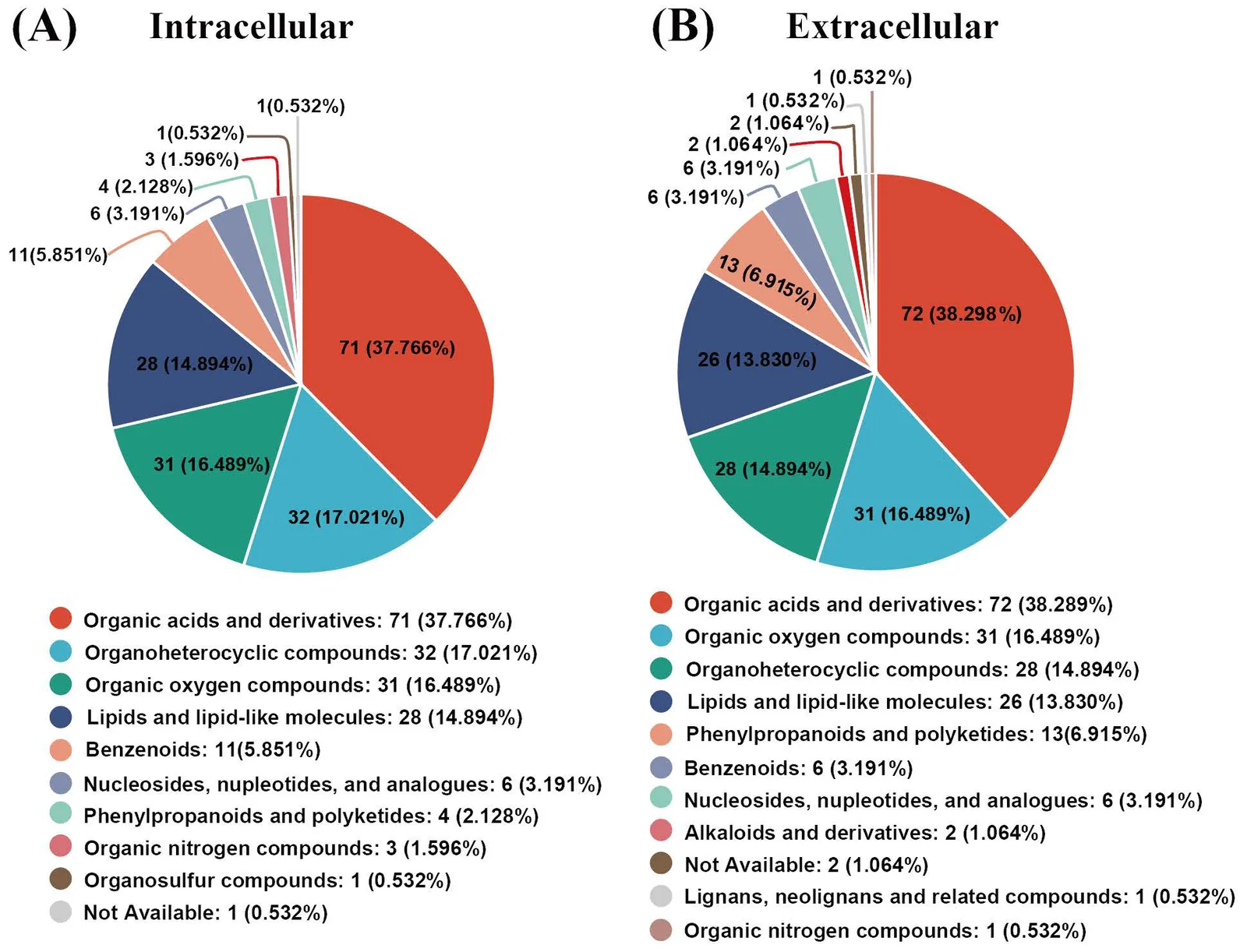
Chemical classification and proportional distribution of differential metabolites in Hs68 cells. (**A**) Classification of intracellular differential metabolites. (**B**) Classification of extracellular differential metabolites.

To further characterize the chemical composition of these differential metabolites, we performed classification analysis based on the Human Metabolome Database (HMDB). In both intracellular and extracellular metabolomes, organic acids and derivatives were predominant, constituting approximately 38% of all differential metabolites, followed by organoheterocyclic compounds, organic oxygen compounds, and lipids and lipid-like molecules (Figure 8A,B). The overall chemical classification of differential metabolites was comparable between the intracellular and extracellular metabolomes.

### 3.8. KEGG Pathway Analysis of Differential Metabolites in the Intracellular and Extracellular Metabolome

**Figure 9:**
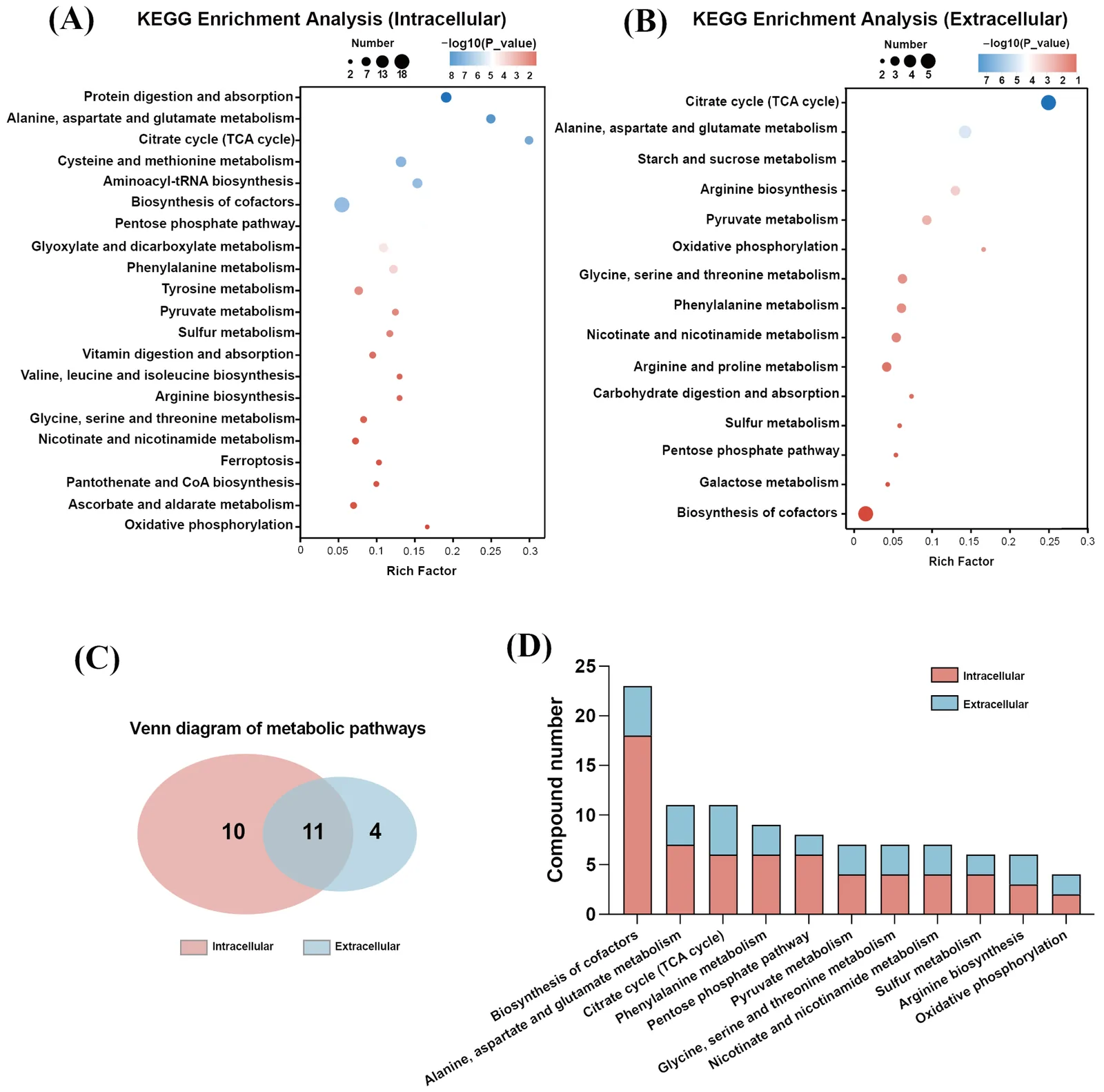
KEGG pathway enrichment analysis of differential metabolites upregulated by BL-G101 relative to the UVB group. (**A**) Enriched pathways in intracellular upregulated metabolites. (**B**) Enriched pathways in extracellular upregulated metabolites. (**C**) Overlap of enriched pathways between intracellular and extracellular metabolomes. (**D**) Differential metabolite counts for the 11 overlapping pathways.

In order to elucidate the biological pathways of the differential metabolites, we performed a KEGG pathway enrichment analysis. The results indicated that, compared to UVB group, the differential intracellular metabolites upregulated in BL-G101 group were enriched in the following signaling pathways (Figure 9A): protein digestion and absorption, alanine, aspartate and glutamate metabolism, TCA cycle, cysteine and methionine metabolism, aminoacyl-tRNA biosynthesis, biosynthesis of cofactors, pentose phosphate pathway, glyoxylate and dicarboxylate metabolism, phenylalanine metabolism, tyrosine metabolism, pyruvate metabolism, sulfur metabolism, vitamin digestion and absorption, valine, leucine and isoleucine biosynthesis, arginine biosynthesis, glycine, serine and threonine metabolism, nicotinate and nicotinamide metabolism, ferroptosis, pantothenate and CoA biosynthesis, ascorbate and aldarate metabolism, and oxidative phosphorylation.

In the extracellular metabolome (Figure 9B), the differential metabolites upregulated in the BL-G101 group compared with UVB group were enriched in the TCA cycle, alanine, aspartate and glutamate metabolism, starch and sucrose metabolism, arginine biosynthesis, pyruvate metabolism, oxidative phosphorylation, glycine, serine and threonine metabolism, phenylalanine metabolism, nicotinate and nicotinamide metabolism, arginine and proline metabolism, carbohydrate digestion and absorption, sulfur metabolism, pentose phosphate pathway, galactose metabolism, and biosynthesis of cofactors.

Given the substantial overlap in enriched pathways between the intracellular and extracellular metabolomes, we next identified pathways commonly regulated in both compartments. As shown in Figure 9C, 11 metabolic pathways overlapped between the intracellular and extracellular metabolomes. Among these shared pathways, the biosynthesis of cofactors pathway contained the largest number of differential metabolites (Figure 9D). Further comparison of this pathway between the BL-G101 and UVB groups revealed 18 significantly altered intracellular metabolites, including vitamins (folic acid, thiamine, biotin, and pantothenic acid) and amino acids (L-aspartic acid, L-methionine, L-tyrosine, L-glutamic acid, and L-tryptophan) (Table 1). In the extracellular metabolome, five metabolites within this pathway were significantly increased, all of which were also upregulated intracellularly (Table 2).

Collectively, these findings indicate that BL-G101 treatment upregulates metabolic pathways related to the biosynthesis of cofactors and several amino acid metabolisms. These pathway alterations suggest a potential link to the anti-photoaging effects of BL-G101.

## 4. Discussion

Here, UVB was employed as the light source to induce photoaging in human Hs68 dermal fibroblasts. UV radiation consists primarily of three types: UVA, UVB, and UVC. Among these, UVA and UVB are considered to reach Earth’s surface and have pathophysiological significance. Compared with UVA (320–400 nm), UVB (280–320 nm) contains more energy and is therefore more likely to cause skin damage [20]. When UV light irradiates the skin, UVB is absorbed within the epidermis and can also pass through this layer to reach the papillary dermis [21,22]. UVB irradiation could modulate various signal pathways, leading to reduced ECM content and contributing to structural unevenness and skin collapse [3]. In the dermis, the ECM is synthesized and secreted by dermal fibroblasts and serves a key function in preserving skin structure and homeostasis [23,24]. Due to the critical role in skin, monolayer cultures of human dermal fibroblasts represent a widely adopted model to explore the cellular mechanisms behind skin aging [20,25]. Our findings indicated that relative to untreated controls, exposure of human Hs68 dermal fibroblasts to 5 mJ/cm^2^ UVB significantly induced ECM loss (reductions in collagen and hyaluronic acid levels), and increased oxidative stress, inflammation, and senescent cells (Figure 2, Figure 3, Figure 4 and Figure 5). Thus, we employed an in vitro model of Hs68 cells exposed to UVB irradiation to evaluate the anti-photoaging effects of two strains, BL-G101 and LA-G80.

Upon exposure to UV radiation, the natural cutaneous antioxidant defense is impaired, leading to the excessive production of free radicals [26]. Such overproduction disturbs REDOX balance, as evidenced by the inhibition of antioxidant enzyme activity, such as SOD. SOD is an endogenous antioxidant enzyme that scavenges free radicals and contributes to maintaining the cellular REDOX system [27]. The levels of SOD serve as an indirect indicator of radical scavenging activity. Excessive free radicals also lead to lipid oxidation, resulting in the production of MDA, a byproduct of oxidative stress. A pattern of reduced SOD activity accompanied by elevated MDA content reflects a pronounced oxidative stress state [28]. Certain natural products and probiotic strains have been reported to attenuate skin photoaging, in part through the alleviation of oxidative stress [29]. In the present study, BL-G101 treatment reduced MDA levels and increased SOD activity in the UVB-exposed Hs68 cells, indicating that the protective effects of BL-G101 against photoaging are associated with improved cellular oxidative stress in dermal fibroblasts.

SA-β-gal serves as the most common biomarker of aging and is still the gold standard for identifying senescent cells. In the present study, BL-G101 treatment significantly decreased the percentage of SA-β-gal-positive cells, suggesting a decline in the senescent fibroblast population. Senescent cells promote a chronic low-grade inflammatory state, often referred to as “inflammaging,” which is associated with aging in various tissues [30,31]. TNF-α and IL-1β are important inflammatory cytokines associated with cellular senescence and UVB-induced inflammatory responses. These inflammatory cytokines could trigger downstream signaling pathways that promote ECM degradation and further accelerate accumulation of senescent cells [32,33]. Our results demonstrate that BL-G101 treatment significantly inhibited the elevation of UVB-induced TNF-α and IL-1β levels. Taken together, these findings suggest that the protection against UVB-induced skin damage by BL-G101 involves reducing levels of pro-inflammatory cytokines and decreasing the number of senescent cells.

According to KEGG pathway enrichment analysis, our results revealed that the biosynthesis of cofactors pathway was identified as one of the significantly enriched pathways in both compartments following BL-G101 treatment of UVB-exposed Hs68 fibroblasts (Figure 9D). The differential intracellular metabolites upregulated in the BL-G101 group compared with the UVB group included several B vitamins (folic acid, thiamine, biotin, and pantothenic acid) (Table 1), many of which have been reported to be associated with skin health. For instance, folic acid has been reported to possess antioxidant properties and to ameliorate UV-induced DNA damage in human fibroblasts [34,35]. A pantothenate-producing *Rothia kristinae* BF00107 strain was reported to suppress inflammation and restore ECM homeostasis in UVB-exposed fibroblasts [36]. Biotin and thiamine have also been reported to be related to skin health, where they modulate inflammatory responses and promote wound healing [37]. Notably, mammalian cells, including Hs68 fibroblasts, cannot synthesize folate, biotin, or thiamine de novo and therefore depend on dietary intake or microbial sources of these vitamins [38]. Several *B. animalis* strains, such as *B. animalis* subsp. *lactis* F 200 and *B. animalis* subsp. *lactis* BB-12, have been reported to possess varying capacities for B vitamin biosynthesis and to produce folate and thiamine [39,40]. These findings raise the possibility that the observed increased intracellular levels of these B vitamins may be due to uptake or transport by Hs68 fibroblasts.

Amino acids are essential for protein synthesis and serve a critical role in defending cells against UV-induced damage [41,42]. Previous studies have demonstrated that glutamine can enhance procollagen mRNA levels and collagen content in human skin fibroblasts, indicating its importance in skin repair [43]. Additionally, studies have shown the essential amino acid mixture, which includes branched-chain amino acids, arginine, and glutamine, significantly restores dermal collagen synthesis following UVB irradiation in HR-1 hairless mice [44]. Tryptophan administration has been found to inhibit stress-induced cutaneous inflammation and accelerate healing of skin wounds in mice, an effect mediated in part by promoting dermal fibroblast migration through AKT phosphorylation [45]. Moreover, certain strains alleviate the inflammatory response in AD by upregulating tryptophan metabolism, as demonstrated with *Lactobacillus reuteri*, *Lactobacillus salivarius*, and *Bifidobacterium* species [46]. Thus, our findings indicate that BL-G101 may contribute to skin health and UV protection by enhancing the metabolism of vitamins and amino acids within the biosynthesis of cofactors pathway.

Despite these encouraging findings, there remain several limitations to our study. While we investigated the direct effects of BL-G101 on Hs68 dermal fibroblasts, the in vitro fibroblast model does not mimic probiotic ingestion but serves solely as a mechanistic screening model to examine how metabolites secreted by these strains affect cell signaling pathways. Considering that orally ingested microorganisms undergo digestion and absorption in the gastrointestinal tract, low-molecular-weight fractions, bacterial lysates, and other probiotic-derived components should also be considered in future studies. Also, incorporating a positive control in future studies would enable a more comprehensive evaluation of the anti-photoaging efficacy of BL-G101. Further validation in animal models (e.g., SKH-1 hairless mice) and clinical studies is essential to confirm the in vivo anti-photoaging effects and assess potential long-term adverse effects. In light of the important role of these strains in regulating gut microbiota, future investigations could examine whether the observed anti-photoaging benefits are mediated through the gut–skin axis. Additionally, while our data suggest that BL-G101 regulates several metabolic pathways, the specific metabolites responsible for these metabolic shifts remain unidentified. Targeted metabolomic analyses together with in vivo validation will be necessary to define the molecular mechanisms underlying the protective activity of BL-G101.

## 5. Conclusions

In the present study, our findings demonstrate that BL-G101 significantly attenuated UVB-induced reduction in collagen and HA, whereas LA-G80 exhibited no notable effects. Furthermore, BL-G101 treatment reduced UVB-induced oxidative stress, inflammation, and the proportion of senescent fibroblasts. Metabolomic profiling showed that BL-G101 altered intracellular and extracellular metabolic profiles. Differential metabolites were mainly enriched in pathways associated with biosynthesis of cofactors, glycine, serine and threonine metabolism, as well as arginine biosynthesis. Taken together, these findings identify BL-G101 as a promising functional food ingredient for mitigating skin photoaging and promoting dermal health.

## Figures and Tables

**Table 1:** Differential intracellular metabolites involved in cofactor biosynthesis pathways in BL-G101-treated UVB-exposed Hs68 fibroblasts.

Metabolite	VIP	FC	*p*-Value	Regulate (BL-G101 vs. UVB)
Folic Acid	5.1738	2.12	7.83 × 10^−6^	Up
Gulonic Acid	5.0693	1.8035	9.15 × 10^−10^	Up
Udp-A-D-Galacturonic Acid	4.8556	1.8252	0.0001888	Up
L-Aspartic Acid	4.6145	1.9044	5.57 × 10^−7^	Up
Fructose-6-Phosphate	4.5823	1.514	5.56 × 10^−8^	Up
Thiamine	4.1416	1.3622	2.24 × 10^−6^	Up
Isocitric Acid	3.6896	1.5258	9.84 × 10^−6^	Up
Citric Acid	3.6828	1.4206	1.55 × 10^−6^	Up
Biotin	3.6456	1.9676	3.74 × 10^−5^	Up
3-Phosphoglycerate	3.4671	1.3045	6.41 × 10^−8^	Up
Glutamic Acid	2.8995	1.2154	3.60 × 10^−7^	Up
L-Methionine	2.6897	1.1357	8.97 × 10^−7^	Up
Cyclic Pyranopterin Monophosphate	2.2845	1.1883	0.003461	Up
L-Tyrosine	1.7215	1.0514	0.0003798	Up
N’-Formylkynurenine	1.5331	1.1426	0.03362	Up
L-Tryptophan	1.1585	1.0519	0.01338	Up
Pantothenic Acid	1.0925	1.0357	0.0008508	Up
Pyruvic Acid	1.0056	1.0315	0.01741	Up

VIP, variable importance in projection; FC, fold change.

**Table 2:** Differential extracellular metabolites involved in cofactor biosynthesis pathways in BL-G101-treated UVB-exposed Hs68 fibroblasts.

Metabolite	VIP	FC	*p*-Value	Regulate (BL-G101 vs. UVB)
Gulonic Acid	3.8769	1.7198	5.70 × 10^−5^	Up
L-Aspartic Acid	2.9632	1.2967	0.00004126	Up
Isocitric Acid	2.1204	1.1374	0.00001343	Up
Citric Acid	2.1204	1.1374	1.34 × 10^−5^	Up
Fructose-6-Phosphate	1.6637	1.0837	1.47 × 10^−5^	Up

VIP, variable importance in projection; FC, fold change.

## Data Availability

The metabolomics data reported in this paper have been deposited in the OMIX, China National Center for Bioinformation/Beijing Institute of Genomics, Chinese Academy of Sciences (accession no. OMIX020451).

## Abbreviations

The following abbreviations are used in this manuscript:

AD	Atopic dermatitis
BL-G101	*Bifidobacterium animalis* subsp. *lactis* BL-G101
FC	Fold change
HA	Hyaluronic acid
IL-1β	Interleukin-1β
LA-G80	*Lactobacillus acidophilus* LA-G80
MDA	Malondialdehyde
PLS-DA	Partial least squares discriminant analysis
SA-β-gal	Senescence-associated β-galactosidase
SOD	Superoxide dismutase
TNF-α	Tumor necrosis factor-α
UV	Ultraviolet
UVB	Ultraviolet B
VIP	Variable importance in projection

## References

[1] Furman D., Auwerx J., Bulteau A.L., Church G., Couturaud V., Crabbe L., Davies K.J.A., Decottignies A., Gladyshev V.N., Kennedy B.K. (2025). Skin health and biological aging. Nat. Aging.

[2] Geng R., Kang S.G., Huang K., Tong T. (2024). Dietary Isoeugenol Supplementation Attenuates Chronic UVB-Induced Skin Photoaging and Modulates Gut Microbiota in Mice. Nutrients.

[3] Geng R., Kang S.G., Huang K., Tong T. (2021). Boosting the Photoaged Skin: The Potential Role of Dietary Components. Nutrients.

[4] Huang A.H., Chien A.L. (2020). Photoaging: A Review of Current Literature. Curr. Dermatol. Rep..

[5] Tong T., Geng R., Kang S.G., Li X., Huang K. (2024). Revitalizing Photoaging Skin through Eugenol in UVB-Exposed Hairless Mice: Mechanistic Insights from Integrated Multi-Omics. Antioxidants.

[6] Li Z., Lu F., Zhou F., Song D., Chang L., Liu W., Yan G., Zhang G. (2025). From actinic keratosis to cutaneous squamous cell carcinoma: The key pathogenesis and treatments. Front. Immunol..

[7] Geng R., Kang S.-G., Huang K., Tong T. (2025). α-Ionone alleviates chronic UVB exposure-induced skin photoaging in mice by remodeling the gut microbiota. J. Future Foods.

[8] Araya M., Morelli L., Reid G., Sanders M.E., Embarek P.B. (2002). Guidelines for the Evaluation of Probiotics in Food.

[9] Deng K., Fan X., Yuan Z., Li D. (2024). Probiotic effects on skin health: Comprehensive visual analysis and perspectives. Front. Microbiol..

[10] Kim J., Lee Y.I., Mun S., Jeong J., Lee D.G., Kim M., Jo H., Lee S., Han K., Lee J.H. (2023). Efficacy and Safety of Epidermidibacterium Keratini EPI-7 Derived Postbiotics in Skin Aging: A Prospective Clinical Study. Int. J. Mol. Sci..

[11] Dempsey E., Corr S.C. (2022). Lactobacillus spp. for Gastrointestinal Health: Current and Future Perspectives. Front. Immunol..

[12] Cheng J., Laitila A., Ouwehand A.C. (2021). Bifidobacterium animalis subsp. lactis HN019 Effects on Gut Health: A Review. Front. Nutr..

[13] Huuskonen L., Lyra A., Lee E., Ryu J., Jeong H., Baek J., Seo Y., Shin M., Tiihonen K., Pesonen T. (2022). Effects of Bifidobacterium animalis subsp. lactis Bl-04 on Skin Wrinkles and Dryness: A Randomized, Triple-Blinded, Placebo-Controlled Clinical Trial. Dermato.

[14] Zhao J., Kui L., Huang J., Deng J., Liu L., Zhu C., Shi Y., Li C., Xiao Y., Yu J. (2025). Bifidobacterium animalis subsp. Lactis BX-BC08 modulates gut microbiota and secretes alpha-Ketoglutaric acid to alleviate MC903-induced atopic dermatitis. J. Transl. Med..

[15] Im A.R., Lee B., Kang D.J., Chae S. (2018). Skin Moisturizing and Antiphotodamage Effects of Tyndallized Lactobacillus acidophilus IDCC 3302. J. Med. Food.

[16] Lim H.Y., Jeong D., Park S.H., Shin K.K., Hong Y.H., Kim E., Yu Y.G., Kim T.R., Kim H., Lee J. (2020). Antiwrinkle and Antimelanogenesis Effects of Tyndallized Lactobacillus acidophilus KCCM12625P. Int. J. Mol. Sci..

[17] Sunada Y., Nakamura S., Kamei C. (2008). Effect of Lactobacillus acidophilus strain L-55 on the development of atopic dermatitis-like skin lesions in NC/Nga mice. Int. Immunopharmacol..

[18] Geng R., Kang S.G., Huang K., Tong T. (2023). alpha-Ionone protects against UVB-induced photoaging in epidermal keratinocytes. Chin. Herb. Med..

[19] Li M., Kang S.G., Huang K., Tong T. (2025). Streptococcus salivarius subsp. thermophilus ST-G30 Prevents Dexamethasone-Induced Muscle Atrophy in C2C12 Myotubes. Nutrients.

[20] Cavinato M., Jansen-Durr P. (2017). Molecular mechanisms of UVB-induced senescence of dermal fibroblasts and its relevance for photoaging of the human skin. Exp. Gerontol..

[21] D’Orazio J., Jarrett S., Amaro-Ortiz A., Scott T. (2013). UV radiation and the skin. Int. J. Mol. Sci..

[22] Lee C.H., Wu S.B., Hong C.H., Yu H.S., Wei Y.H. (2013). Molecular Mechanisms of UV-Induced Apoptosis and Its Effects on Skin Residential Cells: The Implication in UV-Based Phototherapy. Int. J. Mol. Sci..

[23] Huang J., Heng S., Zhang W., Liu Y., Xia T., Ji C., Zhang L.J. (2022). Dermal extracellular matrix molecules in skin development, homeostasis, wound regeneration and diseases. Semin. Cell Dev. Biol..

[24] Jipu R., Serban I.L., Goriuc A., Jipu A.G., Luchian I., Amititeloaie C., Tarniceriu C.C., Hurjui I., Butnaru O.M., Hurjui L.L. (2025). Targeting Dermal Fibroblast Senescence: From Cellular Plasticity to Anti-Aging Therapies. Biomedicines.

[25] Qiang M., Dai Z. (2024). Biomarkers of UVB radiation-related senescent fibroblasts. Sci. Rep..

[26] Wei M., He X., Liu N., Deng H. (2024). Role of reactive oxygen species in ultraviolet-induced photodamage of the skin. Cell Div..

[27] Saxena P., Selvaraj K., Khare S.K., Chaudhary N. (2022). Superoxide dismutase as multipotent therapeutic antioxidant enzyme: Role in human diseases. Biotechnol. Lett..

[28] Liu L., Ma J., Chen W., Zhang J., Zuo W., Wang M., Li J. (2025). UVB-induced HaCat cell damage and Myricaria Paniculata’s molecular effects. Sci. Rep..

[29] Kim H.Y., Lee S., Lim K.M. (2025). Ketoconazole alleviates UVB-induced photoaging by suppressing ROS generation and mitochondrial dysfunction in dermal fibroblasts and ex vivo porcine skin models. J. Dermatol. Sci..

[30] Franceschi C., Bonafe M., Valensin S., Olivieri F., De Luca M., Ottaviani E., De Benedictis G. (2000). Inflamm-aging. An evolutionary perspective on immunosenescence. Ann. N. Y Acad. Sci..

[31] Salminen A., Kaarniranta K., Kauppinen A. (2022). Photoaging: UV radiation-induced inflammation and immunosuppression accelerate the aging process in the skin. Inflamm. Res..

[32] Ansary T.M., Hossain M.R., Kamiya K., Komine M., Ohtsuki M. (2021). Inflammatory Molecules Associated with Ultraviolet Radiation-Mediated Skin Aging. Int. J. Mol. Sci..

[33] Dai Q., Wang Z., Wang X., Lian W., Ge Y., Song S., Li F., Zhao B., Li L., Wang X. (2025). Vorinostat attenuates UVB-induced skin senescence by modulating NF-kappaB and mTOR signaling pathways. Sci. Rep..

[34] Debowska R., Rogiewicz K., Iwanenko T., Kruszewski M., Eris I. (2005). Folic acid (folacin)-New application of a cosmetic ingredient. Kosmet. Med..

[35] Jurzak M., Ramos P., Pilawa B., Bednarek I.A. (2025). Folic Acid as a Molecule Protecting Cells from the Negative Effects of Ultraviolet Radiation-An In Vitro Study. Pharmaceuticals.

[36] Kim H.B., Kim G., Park E., Kim H., Yu B.S., Lee D.G., Park C.H., Jo H., Park H. (2025). Functional Expansion of the Skin Microbiome: A Pantothenate-Producing Rothia Strain Confers Anti-Inflammatory and Photoaging-Protective Effects. Int. J. Mol. Sci..

[37] Kazandjieva J., Bogdanov G., Bogdanov I., Tsankov N. (2026). Vitamins and skin aging. Clin. Dermatol..

[38] Yoshii K., Hosomi K., Sawane K., Kunisawa J. (2019). Metabolism of Dietary and Microbial Vitamin B Family in the Regulation of Host Immunity. Front. Nutr..

[39] Pompei A., Cordisco L., Amaretti A., Zanoni S., Matteuzzi D., Rossi M. (2007). Folate production by bifidobacteria as a potential probiotic property. Appl. Environ. Microbiol..

[40] Yin J., Peng X., Yang A., Lin M., Ji K., Dai X., Huang J., Li L., Feng L. (2024). Impact of oligosaccharides on probiotic properties and B vitamins production: A comprehensive assessment of probiotic strains. Int. J. Food Sci. Technol..

[41] Ling Z.-N., Jiang Y.-F., Ru J.-N., Lu J.-H., Ding B., Wu J. (2023). Amino acid metabolism in health and disease. Signal Transduct. Target. Ther..

[42] Tanveer M.A., Rashid H., Tasduq S.A. (2023). Molecular basis of skin photoaging and therapeutic interventions by plant-derived natural product ingredients: A comprehensive review. Heliyon.

[43] Karna E., Miltyk W., Wolczynski S., Palka J.A. (2001). The potential mechanism for glutamine-induced collagen biosynthesis in cultured human skin fibroblasts. Comp. Biochem. Physiol. B Biochem. Mol. Biol..

[44] Murakami H., Shimbo K., Inoue Y., Takino Y., Kobayashi H. (2012). Importance of amino acid composition to improve skin collagen protein synthesis rates in UV-irradiated mice. Amino Acids.

[45] Bandeira L.G., Bortolot B.S., Cecatto M.J., Monte-Alto-Costa A., Romana-Souza B. (2015). Exogenous Tryptophan Promotes Cutaneous Wound Healing of Chronically Stressed Mice through Inhibition of TNF-alpha and IDO Activation. PLoS ONE.

[46] Huang Y., Chen L., Liu F., Xiong X., Ouyang Y., Deng Y. (2023). Tryptophan, an important link in regulating the complex network of skin immunology response in atopic dermatitis. Front. Immunol..

